# Editorial for Special Issue on Flexible Electronics: Fabrication and Ubiquitous Integration

**DOI:** 10.3390/mi9110605

**Published:** 2018-11-19

**Authors:** Ramses V. Martinez

**Affiliations:** 1 School of Industrial Engineering, Purdue University, 315 N. Grant Street, West Lafayette, IN 47907, USA; rmartinez@purdue.edu; 2 Weldon School of Biomedical Engineering, Purdue University, 206 S. Martin Jischke Drive, West Lafayette, IN 47907, USA

Based on the premise “anything thin is flexible”, the field of flexible electronics has been fueled from the ever-evolving advances in thin-film materials and devices. These advances have been complemented by new integration processes that enable the fabrication of bendable, conformable, and stretchable electronic devices over large areas using scalable manufacturing processes. As a result, flexible electronics has underpinned much of the technological innovation in the fields of sensors, solar energy, and displays over the last decades. This Special Issue focuses on the numerous challenges that researchers and engineers must overcome to bring flexible electronic solutions to healthcare, environmental monitoring, and the human–machine interface. The scientific hurdles to overcome affect the design, fabrication, and encapsulation of the flexible electronic devices, making new approaches to improve these fabrication steps to have an immediate impact in the reliable functioning of these devices upon a large range of strains and bending angles. This Special Issue, therefore, brings us one step closer to the expansion of flexible electronic and optical devices for their ubiquitous integration, the development of new form factors, and the opening up of new markets.

There are 10 papers published in this Special Issue, covering new strategies for a paradigm shift in the design [1,2,3], fabrication [4,5,6,7], and encapsulation [8,9,10] of next-generation flexible systems. Xiao et al. [1] proposed an “island-bridge” strategy to design high-performance stretchable electronics composed of inorganic rigid components so that that can they can be conformally transferred to non-developable surfaces. The design of stretchable electronic devices requires a metric to evaluate their performance. This metric is provided by Plovie et al. [2] to evaluate the performance of stretchable interconnects.

Recent advancements in nanoscale fabrication methods allow the construction of active materials that can be combined with ultrathin soft substrates to form flexible electronics with high performances and reliability. Kang et al. [6] reviewed the most commonly used fabrication methods—involving novel nanomaterials—to make flexible electronics, using application examples of fundamental device components for electronics and applications in healthcare. An alternative, liquid-metal-based soft electronics circuit, termed “droplet circuit” is presented by Ren and Liu [7]. These intrinsically soft circuits can easily match the mechanical impedance of biological tissue and brings significant opportunities for innovation in modern bioelectronics and electrical engineering. A “tunnel encapsulation” strategy is proposed by Leng et al. [8] in order to avoid the typical lack of durability due to stress concentration of flexible interconnects entirely embedded in elastic polymer silicones, such as polydimethylsiloxane (PDMS).

On the application side, these papers have focused on the implementation of flexible systems in healthcare [4,10], photonics [3], and the human–machine interface [9]. Traditional manufacturing approaches and materials used to fabricate flexible epidermal electronics for physiological monitoring, transdermal stimulation, and therapeutics have proven to be complex and expensive, impeding the fabrication of flexible electronic systems that can be used as single-use medical devices. Sadri et al. [4] report the simple, inexpensive, and scalable fabrication of epidermal paper-based electronic devices (EPEDs) using a bench-top razor printer. These EPEDs are mechanically stable upon stretching and can be used as electrophysiological sensors to record electrocardiograms, electromyograms, and electrooculograms, even under water. Following the trend of fabricating disposable flexible electronic devices for healthcare applications, Stier et al. [10] developed an ultra-soft tattoo-like heater that has autonomous proportional-integral-derivative (PID) temperature control. This epidermal device is capable of maintaining a target temperature typical of medical uses over extended durations of time and to accurately adjust to a new set point in process. The rapid expansion of bio-integrated devices requires the development of new adhesives that will ensure the stability of these systems when implemented over soft biological tissues. Yu and Cheng [5], inspired by the remarkable adhesion properties found in several animal species, review recent developments in the field of tunable adhesives, focusing their applications toward bio-integrated devices and tissue adhesives, where strong adhesion is desirable to efficiently transfer vital signals, whereas weak adhesion is needed to facilitate the removal of those systems.

Tang et al. [3] developed a flexible thermo-optic variable attenuator based on long-range surface plasmon-polariton (LRSPP) waveguide for microwave photonic applications. This flexible plasmonic variable attenuator constitutes a step forward towards the fabrication of high-density photonic integrated circuits and a new solution for data transmission and amplitude control in microwave photonic systems. To improve human–machine interfaces through the construction of neuromorphic computing systems capable of mimicking the bio-synaptic functions, Wang et al. [9] developed a flexible artificial synaptic device with an organic functional layer. This flexible device exhibits retention times of the excitatory and inhibitory post-synaptic currents longer than 60 s.

I would like to take this opportunity to thank all the authors for submitting their papers to this Special Issue. I also want to thank all the reviewers for dedicating their time and helping to improve the quality of the submitted papers.
